# Large-scale high-density brain-wide neural recording in nonhuman primates

**DOI:** 10.1038/s41593-025-01976-5

**Published:** 2025-06-23

**Authors:** Eric M. Trautmann, Janis K. Hesse, Gabriel M. Stine, Ruobing Xia, Shude Zhu, Daniel J. O’Shea, Bill Karsh, Jennifer Colonell, Frank F. Lanfranchi, Saurabh Vyas, Andrew Zimnik, Elom Amematsro, Natalie A. Steinemann, Daniel A. Wagenaar, Marius Pachitariu, Alexandru Andrei, Carolina Mora Lopez, John O’Callaghan, Jan Putzeys, Bogdan C. Raducanu, Marleen Welkenhuysen, Mark Churchland, Tirin Moore, Michael Shadlen, Krishna Shenoy, Doris Tsao, Barundeb Dutta, Timothy Harris

**Affiliations:** 1 Department of Neuroscience, Columbia University Medical Center, New York, NY USA; 2 Zuckerman Institute, Columbia University, New York, NY USA; 3 Grossman Center for the Statistics of Mind, Columbia University, New York, NY USA; 4 Department of Neurological Surgery, UC Davis, Davis, CA USA; 5 Meta Reality Labs, Seattle, WA USA; 6 Department of Molecular and Cell Biology, University of California, Berkeley, CA USA; 7 McGovern Institute for Brain Research, Massachusetts Institute of Technology, Cambridge, MA USA; 8 Department of Brain & Cognitive Sciences, Massachusetts Institute of Technology, Cambridge, MA USA; 9 Department of Neurobiology, Stanford University, Stanford, CA USA; 10 Howard Hughes Medical Institute, Stanford University, Stanford, CA USA; 11 Department of Electrical Engineering, Stanford University, Stanford, CA USA; 12 Wu Tsai Neuroscience Institute, Stanford University, Stanford, CA USA; 13 Bio-X Institute, Stanford University, Stanford, CA USA; 14 Janelia Research Campus, Howard Hughes Medical Institute, Ashburn, VA USA; 15 Computation & Neural Systems, California Institute of Technology, Pasadena, CA USA; 16 IMEC, Leuven, Belgium; 17 Kavli Institute for Brain Sciences, Columbia University, New York, NY USA; 18 Howard Hughes Medical Institute, Columbia University, New York, NY USA; 19 Neuroscience Graduate Program, Stanford University, Stanford, CA USA; 20 Department of Bioengineering, Stanford University, Stanford, CA USA; 21 Department of Neurosurgery, School of Medicine, Stanford University, Stanford, CA USA; 22 Howard Hughes Medical Institute, Berkeley, CA USA; 23 Department of Biomedical Engineering, Johns Hopkins University, Baltimore, MD USA

**Keywords:** Sensorimotor processing, Extracellular recording, Motor cortex, Decision

## Abstract

High-density silicon probes have transformed neuroscience by enabling large-scale neural recordings at single-cell resolution. However, existing technologies have provided limited functionality in nonhuman primates (NHPs) such as macaques. In the present report, we describe the design, fabrication and performance of Neuropixels 1.0 NHP, a high-channel electrode array designed to enable large-scale acute recording throughout large animal brains. The probe features 4,416 recording sites distributed along a 45-mm shank. Experimenters can programmably select 384 recording channels, enabling simultaneous multi-area recording from thousands of neurons with single or multiple probes. This technology substantially increases scalability and recording access relative to existing technologies and enables new classes of experiments that involve electrophysiological mapping of brain areas at single-neuron and single-spike resolution, measurement of spike–spike correlations between cells and simultaneous brain-wide recordings at scale.

## Abstract (web summary)

Neuropixels 1.0 NHP is a 45-mm, high-density silicon probe capable of recording large numbers of neurons with single-neuron resolution from most areas in a macaque’s brain.

## Main

High-channel count electrophysiological recording devices such as Neuropixels probes^1,2^ are transforming neuroscience with rodent models by enabling recording from large populations of neurons anywhere in the rodent brain^3–9^. The capabilities provided by this approach have led to a myriad new discoveries^4,7,9^. The Neuropixels 1.0 probe has also been used to record neurons in both human^10,11^ and nonhuman primates (NHPs) such as macaques^12–15^, but its 10-mm length restricts access to superficial targets and the thin shank (24 µm) renders them difficult to insert through primate dura mater. Consequently, a large community of NHP researchers articulated the need for a probe that allows large-scale access to neurons throughout the primate brain.

Current linear array technologies, such as V- or S-probes (Plexon, Inc.), provide access to the whole brain, but are limited to 64 channels and have relatively large diameters (for example, 380 µm) that increase as recording channels are added. Surface arrays such as the Utah array^16^ or floating microwire arrays^17^ allow for recording from up to 256 channels simultaneously, but are limited to recording at pre-specified depths in superficial cortex, require opening the dura for placement and cannot be moved after implantation. Alternative approaches using individually driven single electrodes have achieved recordings in deep brain regions from dozens or hundreds of neurons by a few dedicated labs^18–20^, although these approaches do not allow for dense sampling within a specific target region. Alternatively, two-photon (2P) imaging enables recording at single-cell resolution, but with limited temporal resolution and limited access to many brain areas.

**Fig. 1:**
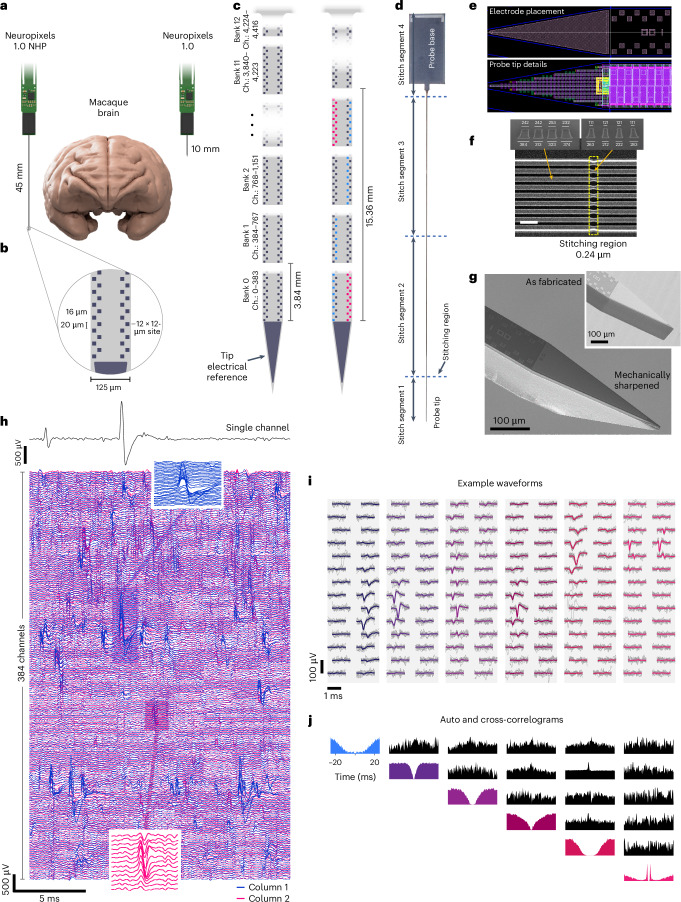
Neuropixels 1.0 NHP probe characterization and engineering. **a**, Comparison of probe geometry with macaque brain and the Neuropixels 1.0 probe. **b**, Recording site layout. **c**, Some 4,416 recording sites covering the full length of the shank, grouped in 11.5 banks of 384 channels. Ch., channel. **d**, Neuropixels 1.0 NHP probe silicon die photograph, indicating the four segments or sub-blocks used for the subfield stitching fabrication process. **e**, Details of the shank tip reference electrode layout (top) and CMOS circuit layout (bottom). The different shapes and colors represent the CMOS process layers used in the layout. **f**, SEM image of one of the metal layers in the shank when crossing the stitching region. Scale bar, 1 µm. Top left, cross-section taken outside the stitching overlap region. Top right, cross-section at the narrowest point. The metal wires are narrower as a result of the double resist exposure. Inset scale bar units in nanometers. **g**, SEM image of shank tip mechanically ground out of plane to 25°. Inset, probe-tip geometry as fabricated, pre-grinding. **h**, Raw electrical recordings from 384 simultaneous channels in the motor cortex of a rhesus macaque monkey. Insets, expanded view of single spike events from single neurons. **i**, Example waveforms from isolated single neurons (gray) and median waveform (colored). **j**, Auto- and CCGs for neurons shown in **i**.

**Extended Data Fig. 1:**
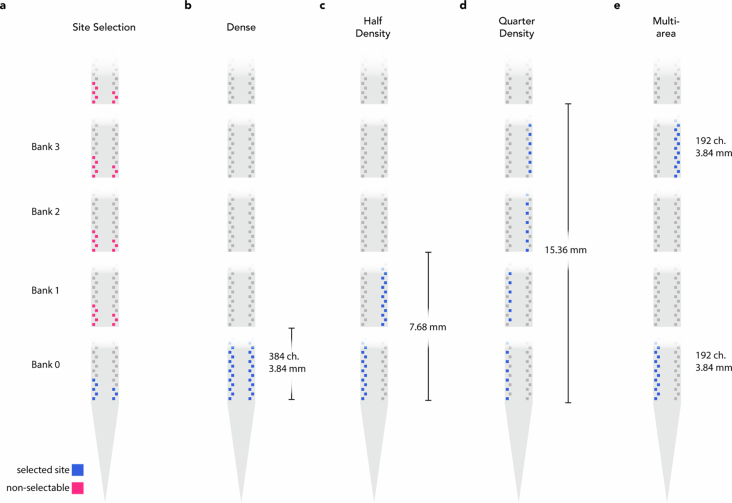
Site selection rules and common configurations. **a**, Site selection rule - any electrode selected on any bank is unavailable for selection in other banks. Multiple electrodes can be connected to a single readout channel, as explored by ref. ^2^, but this impacts the signal to noise and detection for small units, and is a specialized method and not currently in widespread use. **b**, Dense recording from Bank 0, places 384 channels spanning 3.84 mm. **c**, half density configuration covering Banks 0 and 1, spanning 7.68 mm. **d**, Quarter density, covering banks 0–3, spanning 15.36 mm. **e**, multi-area recording.

We developed the Neuropixels 1.0 NHP, a high-density integrated silicon electrode array, optimized for recording in NHPs and designed to enable flexible, configurable recording from large neuronal populations throughout the brain with single-neuron and single-spike resolution. Although the probe’s design and electronic specifications are based on the Neuropixels 1.0 probe (http://Neuropixels.org), fabricating these probes with the desired combination of mechanical and electrical properties using a standard CMOS photolithography process required substantial engineering advances. Each probe is larger than a photolithographic reticle, requiring ‘stitching’ of electrical traces across the boundaries between multiple reticles to achieve the required probe geometry^21^ and the introduction of stress compensation layers to prevent intrinsic material stresses from causing the shank to bend. Relative to Neuropixels 1.0, the two variants of Neuropixels 1.0 NHP feature a longer, wider and thicker shank (45 mm long, 125 µm wide and 90 µm thick; Fig. 1a). For each probe variant, the full length of the shank is populated with recording sites with a density of two sites every 20 µm (Fig. 1b). A switch under every site allows flexible selection of the 384 simultaneous recording channels across these 4,416 sites (11 banks of 384 channels, respectively, plus one half-sized bank at the shank–base junction; Fig. 1c and Extended Data Fig. 1).

Here, we described this new probe and the engineering challenges surmounted by fabrication and illustrate the ability to address a range of new experimental use cases for recording in large animal models. We focused on recording large neuronal populations, recording both in deep structures and from multiple regions simultaneously. We illustrated these use cases with four example experiments from different labs, each pursuing research questions spanning sensory, motor and cognitive domains using macaques. The scale and access provided by Neuropixels 1.0 NHP enabled a wide range of new experimental paradigms, while streamlining neural data collection at a fraction of the cost of existing alternatives.

## Results

### Technology

The Neuropixels 1.0 NHP probe uses the same signal-conditioning circuits as the Neuropixels 1.0 probe^1^, integrating 384 low-noise readout channels with programmable gain and 10-bit resolution on a 130-nm Silicon-on-Insulator CMOS platform. Within the probe shank, an array of shift-register elements and a switch matrix enable programmable selection of recording sites. The probe is fabricated as a 54-mm-long monolithic piece of silicon that integrates both the shank and the base electronics, exceeding the size of the optical reticle used to define on-chip features. We addressed this limitation by developing a method called stitching^21^, which precisely aligns features from different exposures between adjacent reticles.

The typical maximum size of a 130-nm CMOS chip is limited by the maximum reticle size (that is, 22 mm × 24 mm in this case) that can be exposed in a single lithography step. To build larger chips, the CMOS chip is divided into sub-blocks smaller than the reticle field, which are later used to re-compose (that is, stitch together) the complete chip by performing multiple reticle exposures per layer. The stitched boundary regions of the small design blocks are overlapped (that is, double exposed) to ensure uninterrupted metal traces from one reticle to the next, which is accounted for in the design. As multiple levels of metal wires are needed to realize and interconnect the large number of recording sites at high density, the conducting wires in the shank must be aligned between adjacent reticles for four of the six stacked aluminum metal layers. To enable stitching and to simplify photo-mask design and reuse, the probe is designed with three elements as shown in Fig. 1d: a 5-mm tip (Fig. 1e), two 20-mm middle shank segments (stitch segments 2 and 3 in Fig. 1d) and the 10-mm base segment. Four reticles are thus required to define the features on each probe. The probe base is 6 mm wide, allowing four probes to be written across these four reticles.

**Extended Data Fig. 2:**
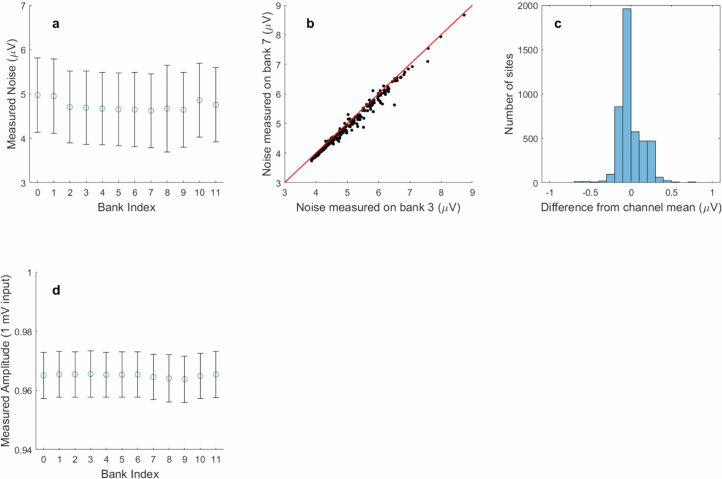
Noise and gain characterization across banks and recording sites. **a**, Distribution of measured noise (mean +/− SD) for 384 recording channels across probe banks 0-11. **b**, Correlation of noise measured on the same readout channel for all recording channels recording from bank 3 (abscissa) and bank 7 (ordinate). Correlation between all pairs of banks is similar. **c**, Distribution of difference of noise recorded from one readout site and other sites that can be connected to the same readout channel. For the probe tested, a total of three sites had measured noise > channel mean + 2 uV. **d**, Measured peak-to-peak amplitude of a 1 mV, 3 kHz sine wave (mean +/− SD) as a function of position along the probe. Additional methodological details provided in the Materials and Methods Summary.

Four design strategies were developed to mitigate the expected degradation of signal with increased shank length: (1) the 384 metal wires connecting recording sites to the readout circuits in the base were widened relative to Neuropixels 1.0 to keep their resistance and thermal noise contribution low; (2) the spacing between the metal wires was increased to limit the signal coupling and crosstalk along the shank; (3) the power-supply wires connected to shank circuits were made wider to minimize voltage attenuation and fluctuations along the shank; and (4) the size of the decoupling capacitors was increased. The additional width and spacing of the metal lines also mitigated the impact of anticipated reticle misalignments, magnification and rotational errors in the overlap regions during stitching. Collectively, these approaches successfully enabled recording from the full 45 mm without signal degradation as a function of position (Extended Data Fig. 2).

Figure 1e shows details of the shank recording site placement and the dense CMOS layout in the shank and Fig. 1f shows a scanning electron microscope (SEM) image of the aluminium metal wires running along one of the 0.24-µm stitching regions in the shank. As a result of the double exposure of the masking photoresist at the stitching region, the wires became 24–54% narrower in this region, but remained continuous circuits without interruption.

One of the major design changes to this device, compared with the rodent probe^1^, was to strengthen and thicken the probe shank, which was necessary both to support the longer 45-mm shank length and to allow the probe to penetrate primate dura. To achieve this, we increased the thickness of the shank from 24 µm to 90 µm. In addition, as the increased thickness altered the bending profile of the shank, we added stress compensation layers, which help to reduce intrinsic stresses within the material in the shank and enabled us to keep bending within the same range as the rodent probes, despite the 450% increase in length.

For the data reported in the present study, we used the Neuropixels 1.0 NHP probe version with a 125 µm wide, 45 mm long shank, 4,416 selectable recording sites (pixels) and a 48 mm^2^ base. To facilitate insertion of the shank into the brain and to minimize dimpling and tissue damage^1^, the 20° top-plane, chisel-tapered shanks were mechanically ground to a 25° bevel angle on the side plane using a modified pipette microgrinder (Narishige, cat. no. EG-402). This procedure resulted in a tip sharpened along both axes (Fig. 1g). Additional discussion of insertion mechanics, methods and hardware is provided in a wiki (https://github.com/cortex-lab/neuropixels/wiki).

### Scientific applications

The programmable sites of Neuropixels 1.0 NHP provide a number of advantages over existing recording technologies appropriate for primates. First, the high-channel count represents a transformative capability for large animal research. Large-scale recordings permitted rapid surveys of brain regions, enabled analyses that infer the neural state on single trials, made it practical to identify ensembles of neurons with statistically significantly correlated spike trains and reduced the number of animals and time required to perform experiments. Second, the high density of recording sites enabled high-quality, automated spike sorting^22^ when recording from one or both columns within a 3.84-mm bank of recording sites (Fig. 1c). The high density of sites also enabled continuous tracking of neurons in the event of drifting motion between the probe and tissue^10,11^, which is imperative for automated spike sorting of most real-world data^22^.

Third, users could choose to record with full density from one column in each of two banks, for 7.68 mm total length of high-quality, single-unit recordings, or specify alternative layouts (Extended Data Fig. 1). Programmable site selection allowed experimenters to decouple the process of optimizing a recording location from probe positioning. This capability allowed experimenters to survey neural activity along the entire probe length to map the relative positions of electrophysiological features. In addition, the programmability allowed experimenters to leave the probe in place before an experiment to improve stability during the recording.

We illustrated these collective advantages using example recordings in different macaque brain studies, including: (1) retinotopic organization of extrastriate visual cortex; (2) neural dynamics throughout the motor system; (3) face recognition in face patches of the inferotemporal (IT) cortex; and (4) neural signals underlying decision-making in the posterior parietal cortex.

#### Dense recordings throughout the primate visual cortex

**Fig. 2:**
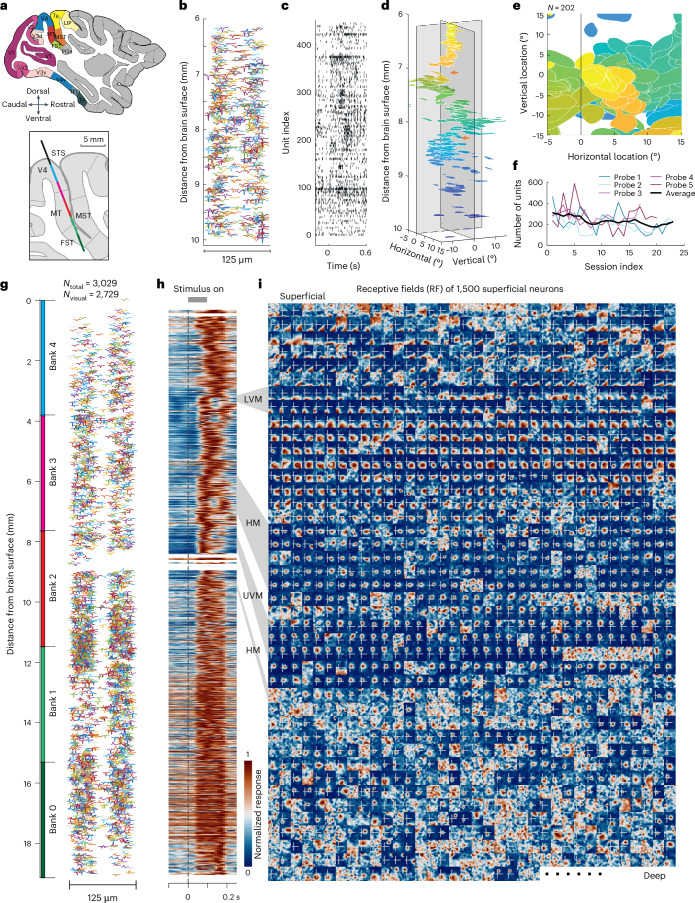
Single- and multi-bank recordings across multiple visual cortical areas. **a**, Visual areas within macaque neocortex shown in a sagittal section. Inset, the estimated probe trajectory of one multi-bank recording. **b**, Spike waveforms of single neurons recorded across a single bank of (384) recording sites (3.84 mm) shown at their measured location on the probe surface. **c**, Population spike raster aligned to stimulus onset for units shown in **b**. **d**, Distribution of RFs of 202 visually responsive neurons across cortical depth in a single-bank recording. The arrows denote abrupt changes in RF progressions and putative visual area boundaries. **e**, Top view of **c**, illustrating the coverage of RFs across the contralateral visual field. **f**, The number of units identified by Kilosort 2.0 for each of the five probes recorded in the same location. Each probe was repeatedly used for up to 23 successive sessions. Units with firing rate >3 Hz are included. **g**, Spike waveforms of 3,029 single neurons recorded across 5 banks of recording sites (~19 mm) shown at their measured location on the probe surface. **h**, Heatmap of stimulus-evoked responses for all 2,729 visually responsive neurons. Each neuron is plotted at its corresponding cortical depth. The dashed black line denotes stimulus onset and the gray line at the top the 0.1-s duration. The gray shading on the right denotes depths where RFs fell on the LVM, HM or UVM. **i**, RF heat maps for 1,500 of the most superficial neurons, indicating visual field locations where stimuli evoked responses for each single neuron. The white crosshair in each map denotes the estimated horizontal and vertical meridians, with each map covering 26 (H) × 32 (V) d.v.a. RFs are arranged in a 42 (rows) × 36 (columns) array. *N*_total_, total number of neurons; *N*_visual_, number of visually responsive neurons.

More than half of the macaque neocortex is visual in function^23^ and a multitude of visual areas containing neurons with distinct feature-selective properties (for example, motion, color) lie beyond the primary visual cortex (V1). However, many of these visual areas are located deep within the convolutions of the occipital, temporal and parietal lobes (Fig. 2a). This, combined with the limitations of prior recording technologies, led to most electrophysiological studies focusing on only a subset of visual areas. Fewer than half of the identified visual areas have been well studied (for example, areas V4, MT (middle temporal)), whereas most (for example, DP (dorsal prelunate), V3A, FST (fundus of the superior temporal sulcus), PO (parieto-occipital)) have been only sparsely investigated. Given the clear similarities between the macaque and human visual systems^24,25^, systematic investigation across the macaque visual cortex is needed. This is practical only with technologies that enable large-scale surveys via simultaneous population recordings from both superficial and deep structures. Our initial tests with the Neuropixels 1.0 NHP probe demonstrate that it is well suited for that purpose.

**Extended Data Fig. 3:**
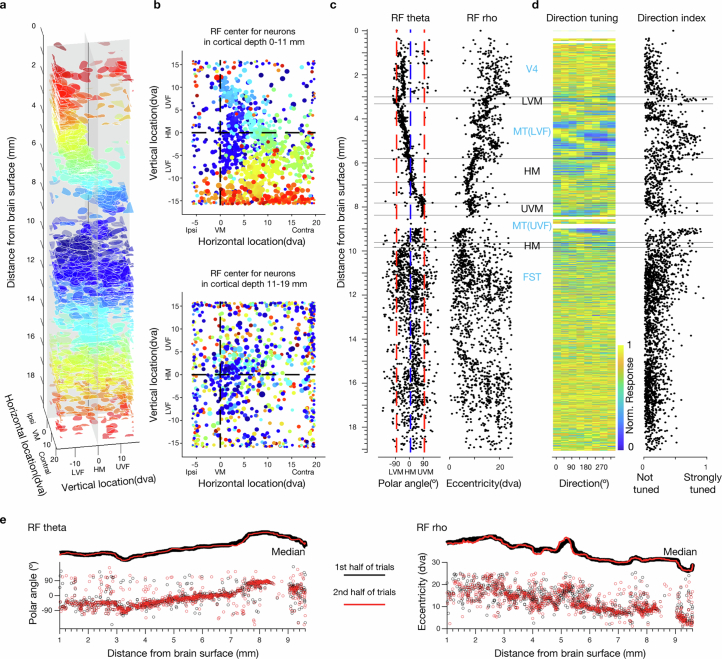
Retinotopic organization and functional properties of single neurons across multiple visual areas. **a**, Distribution of receptive fields (RFs) of 2729 visually responsive neurons across cortical depth from the 5-banks recording across multiple visual areas. Color scale represents cortical depth. **b**, Top-view of **a**, illustrating the progression of RFs across visual fields. RFs from the superficial and deeper part of the brain are demonstrated separately for clarity. **c**, Polar angle (theta) and Eccentricity (rho) of each RF’s geometric center across cortical depth. **d**, Left, heat map of evoked responses across drift directions of grating (vertical thickness is greater for less dense neuronal population). Color scale represents the magnitude of evoked responses. Right, direction index as quantified by the differences of responses to the preferred and its opposite direction divided by the sum of the two. In **c** and **d**, each neuron is plotted at its corresponding cortical depth. Horizontal lines denote the section of cortex where the center of RFs falls on Lower vertical meridians (LVM), horizontal meridian (HM), upper vertical meridian (UVM), and horizontal meridian (HM), respectively, superficial to deep. Putative visual areas are identified and labeled. LVF: lower visual field; UVF: upper visual field; FST: fundus of the superior temporal (FST) area. **e**, Polar angle (theta) and Eccentricity (rho) of each RF’s geometric center across cortical depth calculated using the first and second half of trials within a session, demonstrating stability of function properties of neurons recorded throughout a session.

During individual experimental sessions, the activity of thousands of single neurons across multiple visual cortical areas could be recorded using a single NHP probe. Figure 2b,c shows the spike waveforms and rasters from one recording of 446 neurons simultaneously recorded from one bank of recording sites spanning 6–10 mm below the cortical surface. Of these neurons, 202 neurons exhibited well-defined receptive fields (RFs). As anticipated, the location of the neurons’ RFs varied as a function of the position along the probe. RFs shifted in an orderly manner for stretches of approximately 1 mm and then shifted abruptly, reflecting probable transitions between different retinotopic areas (for example, refs. ^26–28^; Fig. 2d and Extended Data Fig. 3a). Across the full depth, RFs tiled much of the contralateral hemifield and included some of the ipsilateral visual space (Fig. 2e and Extended Data Fig. 3b). When recording from up to 23 successive sessions in the same location with each probe, the number of neurons recorded varied but showed no clear decline with repeated penetrations (Fig. 2f).

In other sessions, we recorded from up to five probe banks spanning 0–19 mm beneath the pial surface (Fig. 2g) across separate blocks of trials. Using this approach, it was possible to sample from different locations without moving the probe. In one example session, 3,029 single neurons were recorded, of which 2,729 neurons were visually responsive (Fig. 2h). As with the single-bank recordings (Fig. 2d,e), neuronal RFs shifted gradually for contiguous stretches, punctuated by abrupt changes. In the example shown (Fig. 2i), RFs at more superficial sites (0–3 mm) were located at more eccentric locations of the visual field and then abruptly shifted toward the center and closer to the lower vertical meridian (LVM; ~3 mm). At the same location, neurons became more selective to the direction of motion (Extended Data Fig. 3c,d), suggesting a transition from area V4 to areas MT or MST. After that, RFs were located more centrally at the lower contralateral visual field and were observed across several millimeters. At deeper sites (~6–7 mm), smaller RFs clustered near the horizontal meridian (HM) for more than 1 mm, then quickly shifted toward the upper vertical meridian (UVM; ~8 mm). Finally, at the deepest sites (>10 mm), RFs generally became larger and much less well defined. These representations were stable throughout the duration of a session (Extended Data Fig. 3e). These data illustrate how the Neuropixels 1.0 NHP’s dense sampling and single-unit resolution facilitate large-scale and unbiased mapping of the response properties of neurons across multiple visual areas.

### Stable, large-scale recording during motor behaviors

Next, we demonstrated the utility of this technology for studying multiple brain areas involved in movement control. Primary motor cortex (M1) is situated at the rostral bank of the central sulcus and extends along the precentral gyrus. Sulcal M1 contains the densest projections of descending corticomotoneuronal cells and corticospinal neurons, which collectively are understood to convey the dominant efferent signals from the brain to the periphery in primates^29,30^. Constraints of existing technology have led to two broad limitations in studies of the motor system.

First, motor electrophysiologists have been forced to choose between simultaneous recording from populations of superficial neurons in gyral motor cortex (PMd and rostral M1) using Utah arrays^31^ and, alternatively, recording fewer neurons in sulcal M1 using single-wire electrodes or passive arrays of 16–32 contacts (for example, Plexon S-probes or Microprobes Floating Microwire Arrays^17^). Recording from large populations of neurons in sulcal M1 has not been feasible.

Second, the motor cortex is only one part of an extensive network of cortical and subcortical structures involved in generating movement^29,32^. Many investigations of the motor system focus on M1 and comparatively fewer experiments have investigated neural responses from the numerous additional structures involved in planning and controlling movements, the result in part of the challenge of obtaining large-scale datasets in subcortical structures in primates. Areas such as the supplementary motor area (SMA) and the basal ganglia (BG) are understood to be important for planning and controlling movements, but investigation of the functional roles and interactions between these regions is hampered by the challenge of simultaneously recording from multiple areas.

**Fig. 3:**
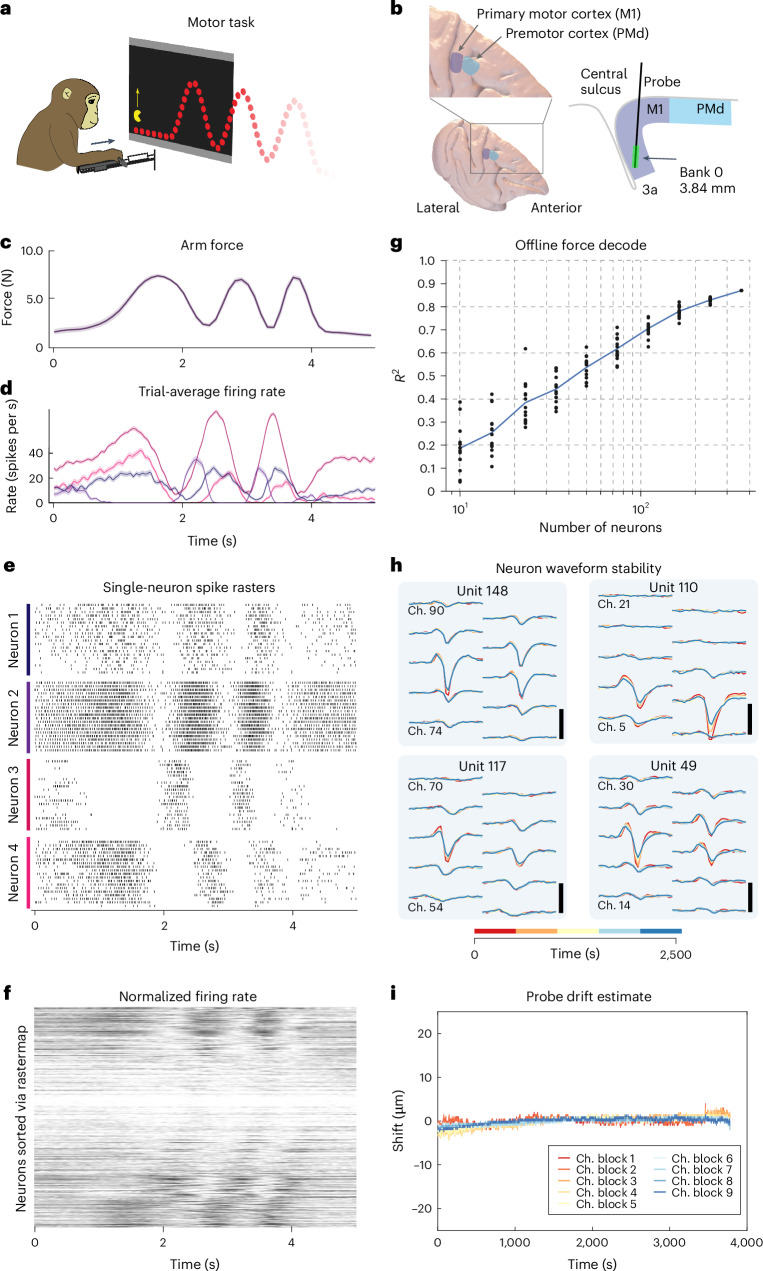
Recording from the rhesus motor system. **a**, Pacman isometric force-tracking task. **b**, Recording targets in motor cortex (left) and schematic of recording target in sulcal M1, sagittal section (right). **c**, Trial-averaged arm force during pacman task. **d**, Trial-averaged firing rate for example neurons. **e**, Single-trial spike raster for four example neurons. **f**, Trial-averaged, normalized responses of all 360 neurons for the same session as data in **c**–**e**, ordered using Rastermap^34^. **g**, Linear model force prediction accuracy as a function of the number of neurons included in the analysis. **h**, Spike waveforms on ten channels of four example neurons, averaged across nonoverlapping time bins that represent one-fifth of the recording duration. **i**, Probe drift estimating output by Kilosort 2.5 over the duration of a single motor behavioral session. Each line represents the estimate for a subset of channels.

We developed a system capable of simultaneous recordings from multiple Neuropixels 1.0 NHP probes in superficial and deep structures of rhesus macaques. We tested this approach using a task in which a monkey generated isometric forces to track the height of a scrolling path of dots (Fig. 3a; task described in ref. ^33^). We recorded from the primary motor and premotor cortex (M1 and PMd; Fig. 3b) while the monkey tracked a variety of force profiles (one profile per condition). Each condition was repeated across multiple trials (Fig. 3c). Single neurons exhibited a diversity of temporal patterns throughout the motor behavior (Fig. 3d–f). When ordered using Rastermap^34^, neurons illustrated a diversity of phase relationships with respect to the behavior (Fig. 3f). Predictions of endpoint force from neural activity (via linear regression) improved steadily as more neurons were included in the analysis and the performance did not saturate even when including all recorded neurons (360) for an example session (Fig. 3g). Thus, despite the apparent simplicity of a one-dimensional force-tracking task, neural responses are sufficiently diverse that it is necessary to sample from many hundreds of neurons to capture a complete portrait of population-level activity.

**Fig. 4:**
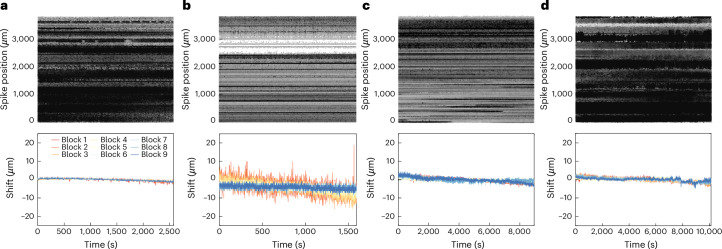
Example drift maps from four brain regions. **a**, Top, drift map visualization of position versus time for one representative recording from the motor cortex. The darker spots indicate larger amplitude spikes. Bottom, estimate of drift calculated using Kilosort for the same recording shown above. **b**–**d**, Same visualization as shown in **a** for visual cortex (**b**), area AF (**c**) and area LIP (**d**), respectively.

**Extended Data Fig. 4:**
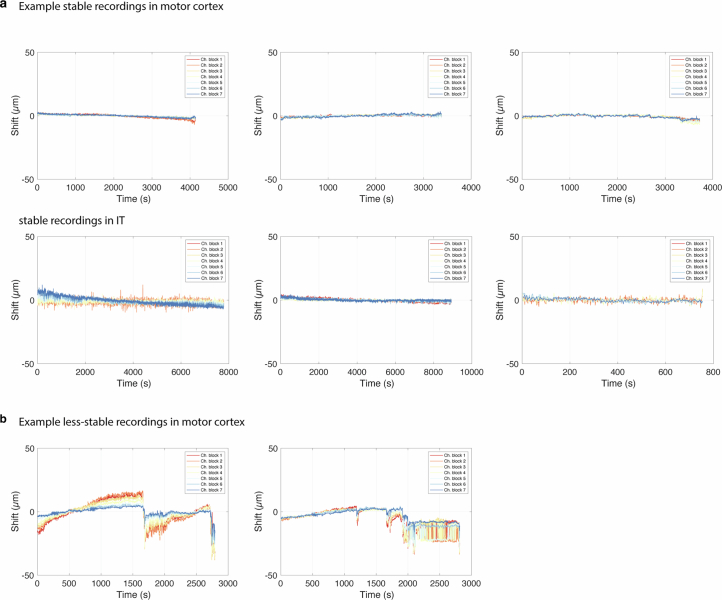
Acute recording stability for multiple sessions. **a**, Probe drift estimates computed using Kilosort 2.5 for six example stable sessions in motor cortex (top) and IT cortex (bottom). **b**, Drift estimates for two less-stable sessions, including both slow drift of the probe within neural tissue and rapid shifts. Unstable sessions were comparably uncommon, and typically resulted from not placing gentle pressure on the surface of dura with a guide tube. Importantly, data from sessions with probe drift can often still be used, as demonstrated by refs. ^4,5^.

**Extended Data Fig. 5:**
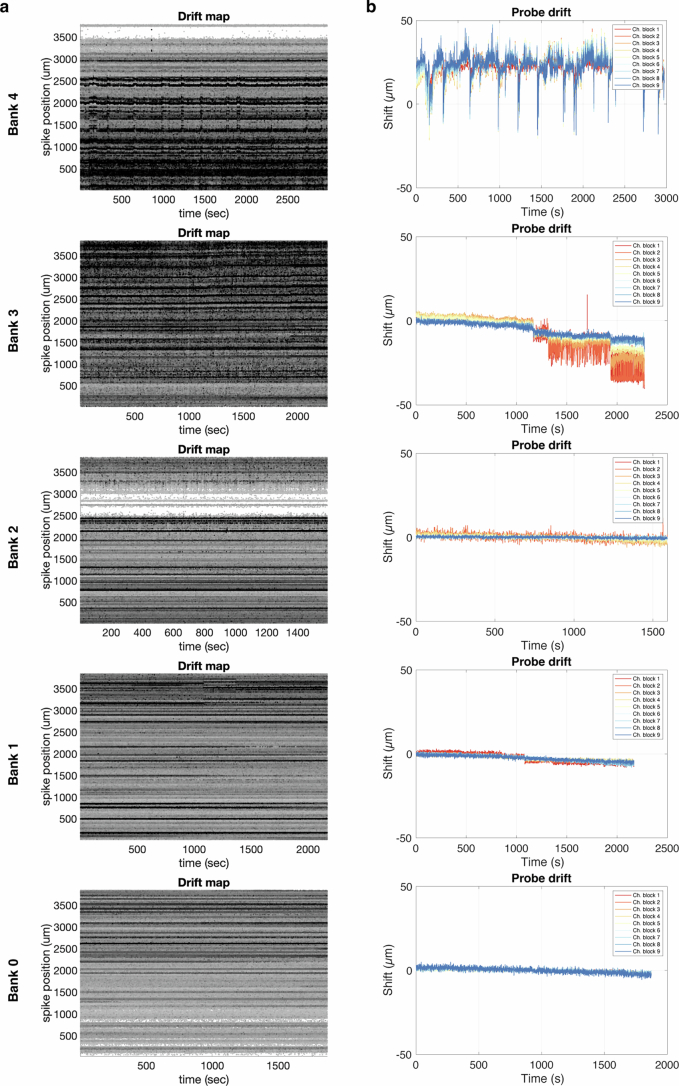
Acute recording stability in visual cortex for multi-bank recordings shown in Fig. 2. Each row shows the drift map for recordings from each of the banks, with bank0 from the deepest of the cortex and bank 4 from the most superficial. **a**, spike raster plot with spikes identified via Kilosort 3 and plotted at estimated depth along the probe. Spikes with larger amplitude are shown with darker colors. **b**, Probe drift estimates. Within each recording, different traces indicate different groups of channels on the probe.

The relative motion of the probe within the brain tissue is a major concern for the stability of neural recordings. Motor tasks represent a challenging set of conditions because movement of the body can place stresses on the implant and move the probe within the brain. With careful preparation, on most sessions we did not observe rapid probe motion due to pulse, respiration or mechanical forces from the motor task. Typically, we observed stable waveforms (Fig. 3h) and ~2–15 µm of slow drift over the course of a recording session (Fig. 3i and Extended Data Figs. 4a and 5). In some isolated cases, when the primate generated a large unexpected movement, we did observe a fast translation of the probe (for example, Extended Data Fig. 4b). However, in practice, we found that low-drift recordings could be obtained across a variety of experimental preparations in disparate brain regions (Fig. 4a–d and Extended Data Figs. 4 and 5).

**Fig. 5:**
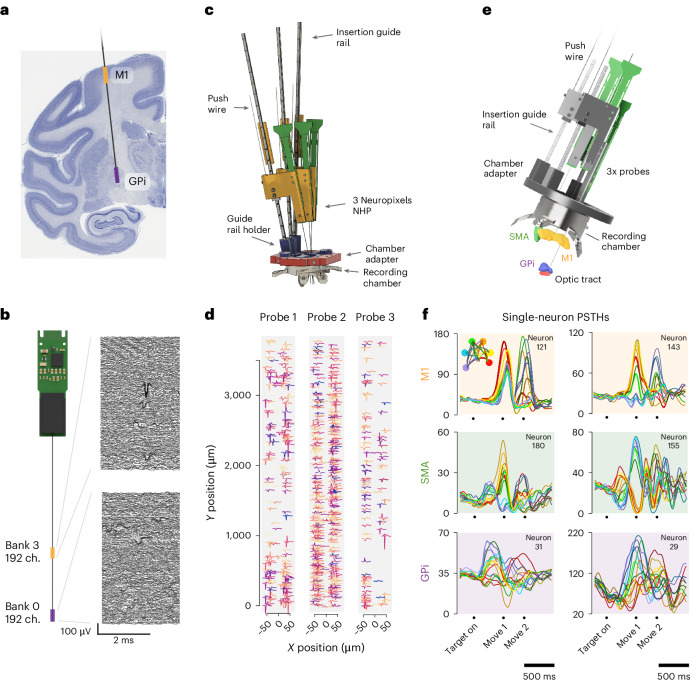
Simultaneous targeting of multiple brain areas with one or more probes. **a**, Multi-area recording using a single probe in the motor cortex and BG (GPi), allocating 192 channels to each region via programmable site selection. **b**, Example raw waveforms and site selection for the recording described in **a**. **c**, Recording from many neurons within a single small target region using multiple probes inserted along convergent trajectories. **d**, Example waveforms of neurons recorded on probes using the apparatus shown in **c**. **e**, Recording from disparate brain regions (SMA, M1 and GPI) using three probes, all inserted along parallel trajectories. **f**, Example neurons recorded using the apparatus shown in **e**.

Programmable site selection in the Neuropixels 1.0 NHP enables simultaneous recording from superficial and deep structures using a single probe. To illustrate this capability, we recorded from superficial motor cortex and internal globus pallidus (GPi) in the BG using 192 channels in each target location (Fig. 5a,b). Alternatively, the small form factor of the Neuropixels 1.0 NHP probes and headstages allows for dense packing of multiple probes by inserting probes along nonparallel trajectories to record from a large number of neurons in a single area. Figure 5c,d illustrates recordings from three probes in gyral PMd, yielding 673 neurons. This approach was tested on two sessions, in which we recorded from 6 and 7 probes, yielding 1,012 and 783 neurons, respectively. These yields were lower than average as a result of imperfect recording conditions unrelated to the insertion hardware. Sampling with multiple probes is also well suited for simultaneous recording from different brain areas. Figure 5e illustrates a system designed to record from M1, the GPi of the BG and the SMA, using multiple probes inserted into a single recording chamber, with representative neural responses illustrated in Fig. 5f.

### Face recognition in the IT cortex

Next, we demonstrated the use of the probes in the inferotemporal (IT) cortex, a brain region in the temporal lobe that is challenging to access because of its depth. The IT cortex is a critical brain region supporting high-level object recognition and has been shown to harbor several discrete networks^35^, each specialized for a specific class of objects. The network that was discovered first and has been most well studied in nonhuman primates is the face-patch system. This system consists of six discrete patches in each hemisphere^36^, which are anatomically and functionally connected. Each patch contains a large concentration of cells that respond more strongly to images of faces than to images of other objects. Studying the face-patch system has yielded many insights that have transferred to other networks in the IT cortex, including increasing view invariance going from posterior to anterior patches and a simple, linear encoding scheme^35^. As such, this system represents an approachable model for studying high-level object recognition^37^. The code for facial identity in these patches is understood well enough that images of presented faces can be accurately reconstructed from neural activity of just a few hundred neurons^38^.

**Fig. 6:**
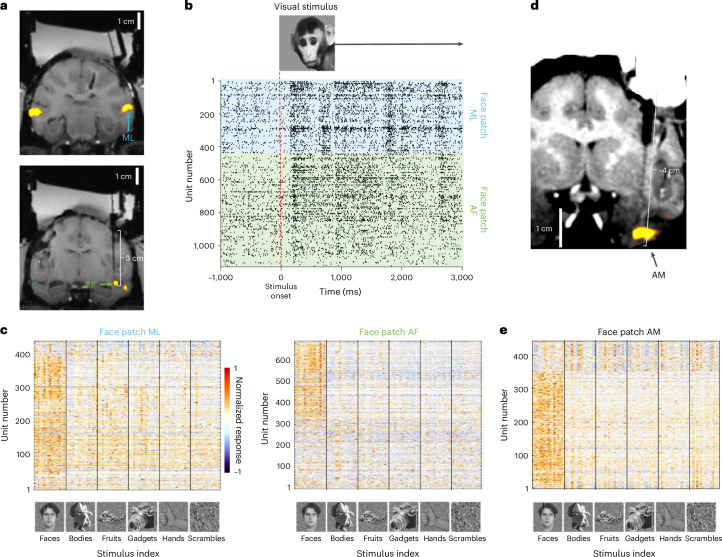
Deep, simultaneous recordings from two face patches in the IT cortex. **a**, Simultaneous targeting of two face patches. Coronal slices from MRI show inserted tungsten electrodes used to verify targeting accuracy for subsequent recordings using Neuropixels 1.0 NHP (top, face-patch ML; bottom, face-patch AF). Yellow overlays illustrate functional MRI contrast in response to faces versus objects. **b**, Response rasters for a single stimulus presentation of simultaneously recorded neurons in ML and AF to a monkey face, presented at *t* = 0. Each line in the raster corresponds to a spike from a single neuron or multi-unit cluster, including both well-isolated single units and multi-unit clusters. **c**, Neuropixels 1.0 NHP enabling recordings from many face cells simultaneously. These plots show the average responses (baseline subtracted and normalized) of visually responsive cells (rows) to 96 stimuli (columns) from 6 categories, including faces and other objects. Bottom, exemplar stimuli from each category. The plots included 438 cells or multi-unit clusters in ML (left) and 689 in AF (right), out of which a large proportion responded selectively to faces. Units were sorted by channel, revealing that face cells are spatially clustered across the probe. **d**, Coronal slice of MRI of Neuropixels probe targeting the deepest IT face-patch AM. The thick shadow is a cannula that was inserted through the dura into the brain. The thin shadow corresponds to the Neuropixels trajectory. **e**, Same as **c**, for face-patch AM, recorded in a different session.

A major remaining puzzle is how different nodes of the face-patch hierarchy interact to generate object percepts. To answer this question, it is imperative to record from large populations of neurons in multiple face patches simultaneously to observe the varying dynamics of face-patch interactions on a single-trial basis. This is essential because the same image can often invoke different object percepts on different trials^39^. In the present study, we recorded with one probe in each of two face patches, middle lateral (ML) and anterior fundus (AF), simultaneously (Fig. 6a). The Neuropixels 1.0 NHP probes recorded responses of 1,127 units (622 single units, 505 multi-units) across both face patches during a single session (Fig. 6a, right). A continuous segment of approximately 220 channels (2.2 mm) in ML and 190 channels (1.9 mm) in AF contained face-selective units, indicating that these extents of the probes were in the IT cortex. In ML 261 units and in AF 297 units were face selective (two-sided, two-sample Student’s *t*-test, threshold *P* < 0.05).

Changing the visual stimulus to a monkey face yielded a clear visual response across both face-patch populations. We measured responses of visually responsive cells to 96 different stimuli containing faces and nonface objects (Fig. 6b,c). A majority of cells in the two patches showed clear face selectivity. Using single-wire tungsten electrodes, this dataset would have taken about 2 years to collect, but is now possible in a single 2-h experimental session^38^. In addition, to the gain in efficiency created by this technology, simultaneous recordings of multiple cells and multiple areas allowed for investigation of how populations encode object identity in cases of uncertain or ambiguous stimuli, where the interpretation of the stimulus may vary from trial to trial but is nevertheless highly coherent on each trial. The anatomical depth of face patches puts them far out of reach for shorter high-density probes. For example, face-patch AM (anteromedial) sits about 42 mm from the craniotomy (Fig. 6d) along a conventional insertion trajectory, but face patches in this region are still accessible using the Neuropixels 1.0 NHP probe and exhibit face-selective responses within the boundaries of AM (Fig. 6e).

### Single-trial correlates of decision-making in LIP

For many cognitive functions, the processes that give rise to behavior vary across repetitions of a task. As such, technologies that enable the analysis of neural activity at single-trial resolution will be especially critical for research on cognition. Achieving this resolution is challenging because it requires recordings from many neurons that share similar functional properties.

**Fig. 7:**
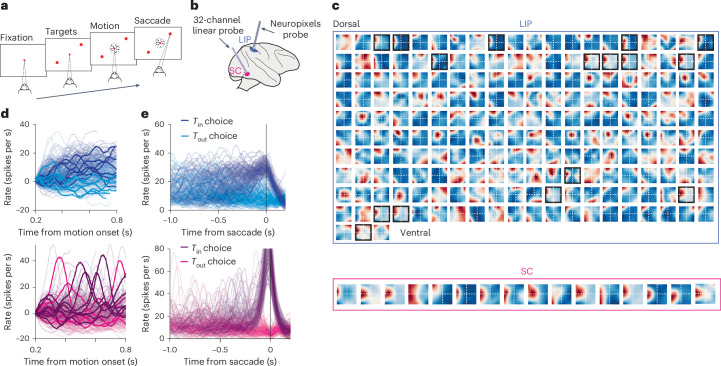
Single-trial dynamics of a decision process in multiple brain regions. **a**, Task. The monkey must decide the net direction of dynamic RDM and indicate its decision by making a saccadic eye movement, whenever ready, from the central fixation point (red) to a left-choice or right-choice target (black). The choice and response times are explained by the accumulation of noisy evidence to criterion level^54^. **b**, Simultaneous recordings in LIP and SC. Populations of neurons were recorded in LIP with a Neuropixels 1.0 NHP probe and in SC (deeper layers) with a multi-channel V-probe (Plexon). **c**, RFs are identified, post-hoc, from control blocks in which the monkey performed an oculomotor delayed response task^55^. A small fraction of the LIP neurons (top) has RFs that overlap the left-choice target (outlined in black). The SC (bottom) has a topographic map, so many neurons have overlapping RFs. The large sample size in LIP facilitates identification of neurons that respond to the same choice target in LIP and SC. **d**, Single-trial activity in LIP (top, *n* = 17) and SC (bottom, *n* = 15). Each trace depicts the smoothed firing rate average (Gaussian kernel, *σ* = 25 ms) of the 17 neurons that overlap the left-choice target (*T*_in_) on a single trial, aligned to the onset of the motion stimulus. Rates are offset by the mean firing rate of 0.18–0.2 s after motion onset to force traces to begin at 0. The line color indicates the decision for the left-choice target or the right-choice target (*T*_out_) on that trial. A few representative traces are highlighted for clarity. **e**, The same trials as in **d** aligned to saccade initiation, without baseline offset. Single-trial firing rates approximate drift diffusion in LIP—the accumulation of noisy evidence—whereas single-trial firing rates in SC exhibit a large saccadic burst at the time of the saccade, preceded by occasional nonsaccadic bursts.

This challenge is exemplified in perceptual decision-making, in which decisions are thought to arise through the accumulation of noisy evidence to a stopping criterion, such that their evolution is unique on each trial in a dynamic motion discrimination task (Fig. 7a; for task see ref. ^40^). This process is widely observed and known as drift diffusion^41,42^. Neural correlates of drift diffusion have been inferred from activity in the lateral intraparietal area (LIP) averaged over many decisions. LIP neurons display spatial RFs and represent the accumulated evidence for directing the gaze toward the RF. Analysis of LIP activity during single decisions has proven particularly difficult because RFs have little to no anatomical organization (Fig. 7c, top), preventing simultaneous recordings from many neurons with the same receptive field.

Neuropixels recording in the LIP overcomes this challenge. These recordings yield 50–250 simultaneously recorded neurons, of which 10–35 share an RF that overlaps one of the contralateral choice targets used by the monkey to report its decision. The average activity of these target-in-RF (*T*_in_) neurons during a single decision tracks the monkey’s accumulated evidence as it contemplates its options. The signal explains much of the variability in the monkey’s choices and reaction times^43^ and conforms to drift-diffusion dynamics (Fig. 7d,e, top).

Neuropixels technology also enables multi-area recordings from ensembles of neurons that share common features (Fig. 7b). For example, neurons in the deeper layers of the superior colliculus (SC) receive input from LIP and, like LIP neurons, also have spatial RFs and decision-related activity (Fig. 7c, bottom). An ideal experiment to understand how the two areas interact is to record simultaneously from populations of neurons in LIP and SC that share the same RF. This experiment is almost impossible with previous recording technology because of the lack of anatomical organization in the LIP.

This second challenge is also overcome by Neuropixels recording in the LIP, allowing for post-hoc identification of neurons in the LIP and SC with overlapping RFs (Fig. 7c). In the experiment depicted in Fig. 5, the response fields of 17 of the 203 LIP neurons overlapped the left-choice target as well as 15 simultaneously recorded neurons in the SC. Unlike LIP, single-trial analysis of the SC population revealed dynamics that are not consistent with drift diffusion (Fig. 7d,e, bottom). Instead, the SC exhibits bursting dynamics, which were found to be related to the implementation of a threshold computation^44^. The distinct dynamics in the LIP and SC during decision-making were observable only through single-trial analyses, the resolution of which is greatly improved with the high yield of the Neuropixels 1.0 NHP recording.

### Measuring spike–spike correlation with high-density probes

Understanding how the anatomical structure of specific neural circuits implements neural computations remains an important but elusive goal of systems neuroscience. One step toward connecting disparate levels of experimental inquiry is mapping correlative measures of relative spike timing between pairs of neurons, which is indicative of either synaptic connection between two neurons or shared input drive^6,13,45^. This is often impractical or extremely challenging when recording from only a small number of neurons, because the likelihood of recording from a pair of neurons with a statistically significant peak in the spike cross-correlogram (CCG) can be quite low. The probability of recording from such pairs of neurons depends on a number of factors, including the details of anatomy in a specific species and brain region. Broadly speaking, however, the probability of successfully recording a pair of neurons with a significant CCG peak increases with the square of the total number of neurons recorded.

**Fig. 8:**
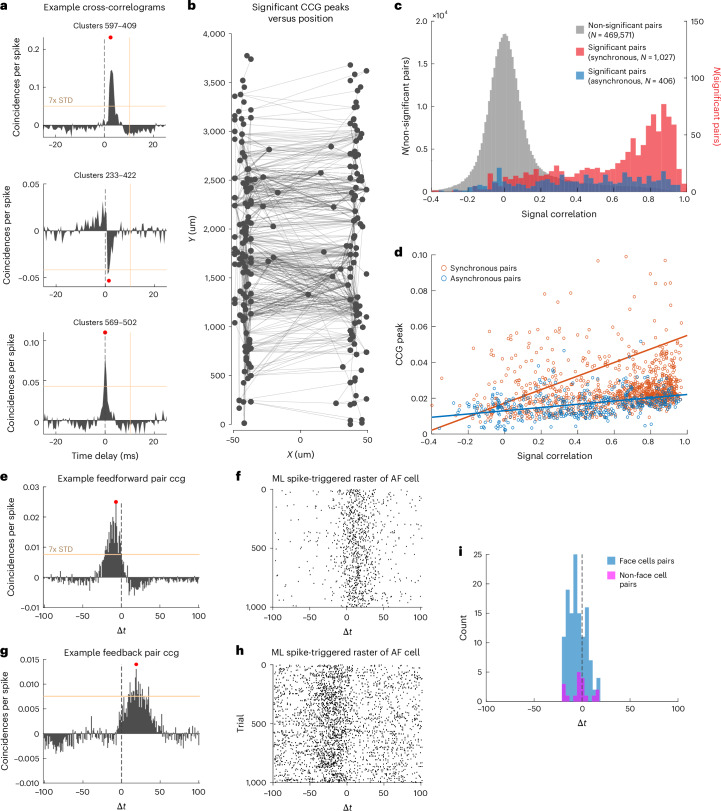
Measuring spike–timing correlations from unit crosscorrelation via high-density recording. **a**, Jitter-corrected CCGs of four example pairs of neurons exhibiting significant correlations in spike timing. 7x STD, 7 × standard deviation of CCG flank. **b**, Diagram of functionally connected neuron pairs from one example session, with neurons ordered by depth along the probe. **c**, Distribution of signal correlation for pairs of neurons with different CCG types in the visual cortex. Group means were compared using bootstrapping between nonsignificant pairs and significant, synchronous pairs (*P* < 10^−4^), between nonsignificant pairs and significant, asynchronous pairs (*P* < 10^−4^) and between synchronous and asynchronous pairs (*P* < 10^−4^). **d**, Relationship between signal correlation and peak value of CCG. **e**,**f**, Example putative connected cell pair identified using two probes in area AF (**e**) and ML (**f**) with putative feedforward connection. **g**,**h**, Example putative connected cell pair identified using two probes in area ML (**g**) and AF (**h**) with putative feedback connection. **i**, The population of functionally connected cells between AL and MF regions dominated by cells that respond to faces.

**Extended Data Fig. 6:**
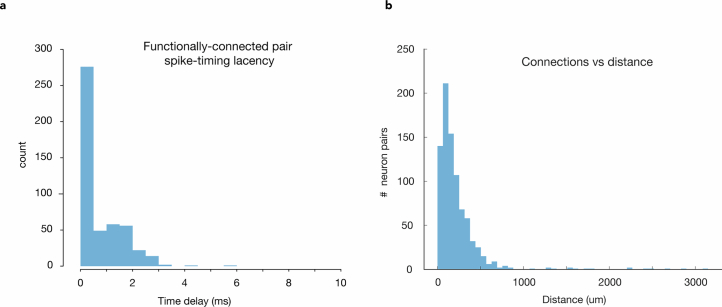
CCG metrics. **a**, Latency distribution between identified peaks in cross-correlograms of neurons with statistically significant spike timing relationships. Example session, monkey C. **b**, Number of pairs of neurons with significant CCG peaks as a function of the distance between two neurons.

The Neuropixels 1.0 NHP probe typically yields 200–450 (and sometimes more) neurons when recording with 384 channels in cortical tissue. Applying the same methodology established in ref. ^6^ to 13 sessions from rhesus PMd and M1 yielded 111 ± 89 putative connected pairs per session and a connection probability of 0.73 ± 0.61%. Figure 8a shows three example jitter-corrected CCG plots between pairs of neurons with significant peaks in the CCG. In many examples the CCG peak lagged between one neuron relative to the other, consistent with a 1- to 2-ms synaptic delay. For other neuron pairs, the CCG peak is synchronous between the two neurons, suggesting that they may receive common input (Fig. 8a, top). Extended Data Fig. 6 illustrates the distribution of spike–timing delays for 479 neurons from an example recording session. Nearby neurons are more likely to exhibit significant CCG peaks than neurons located further apart. Using this approach, we can map the full set of putative connections for a given recording across the cortical lamina (Fig. 8b).

In addition to the cortical distance, we further assessed the dependence of CCG peaks on the tuning similarity between neuronal pairs for the data recorded in the visual cortex. For example, neuron populations with diverse RFs obtained from multiple visual areas (shown in Fig. 2) allow us to quantitatively determine the association between CCG peaks and RF overlap. Using signal correlation (*r*_sig_) as a measure of such tuning similarity in visual fields^46^, we showed that functionally connected neuron pairs exhibit higher *r*_sig_ compared with nonsignificant pairs (Fig. 8c). Specifically, synchronous pairs (putatively receiving common inputs) tend to share highly overlapping RFs (*r*_sig_ mean = 0.60, *P* < 10^−4^ compared with nonsignificant pairs), whereas asynchronous pairs are more likely to share moderately overlapping RFs (*r*_sig_ mean = 0.43, *P* < 10^−4^ compared with nonsignificant pairs and *P* < 10^−4^ compared with synchronous pairs). Moreover, the amplitude of the significant CCG peaks is positively correlated with *r*_sig_ (Fig. 8d), which is consistent with previous studies^47,48^.

This same methodology can be applied to assess CCG peaks between multiple simultaneously recorded regions recorded using separate probes to assess feedforward and feedback signaling between two regions. Figure 8e shows the jitter-corrected CCG for a pair of neurons where one neuron is located in face-patch ML and the other in face-patch AF in the IT cortex. Figure 8f shows a single-trial spike raster of the downstream neuron in area AF, triggered on spikes of the neuron in ML, illustrating a possible feedforward signaling, whereas Fig. 8h illustrates a putative feedback connection with opposite timing response from the cell pair shown in Fig. 8g. Remarkably, pairs of face cells were >10× as likely to have significant peaks in CCG (2.3%, 125 of 5,368 face cell pairs) as pairs where one or both cells were not face cells (0.15%, 18 of 11,912 other pairs) between the ML and AF (Fig. 8i).

## Discussion

We have presented a new recording technology and suite of techniques to enable electrophysiological recordings using high-density integrated silicon probes in rhesus and other nonhuman primates. This technology enables large-scale recordings from populations of hundreds of neurons from deep structures in brain areas that are inaccessible using alternative technologies. The key methodological advance in the Neuropixels 1.0 NHP probe is the longer recording shank, which required the development of techniques to adapt photolithographic silicon-manufacturing methods to allow for stitching across multiple reticles. Creating a long and thin probe shank also required developing approaches for reducing the bending that resulted from internal stresses within the shank.

This technology combines the advantages of multiple approaches—recording with single-neuron spatial resolution and single-spike temporal resolution—whereas the long shank length provides recording access to most of the macaque brain. Programmable site selection enabled recording from multiple brain structures using a single probe, as well as surveying multiple recording sites along the shank without moving the probe. The combination of the compact form factor, commercially available and turn-key recording hardware and modest cost per channel relative to technologies like the Utah array enabled straightforward scaling in the size of simultaneously recorded neural populations. These capabilities could be essential for achieving accurate estimates of neural dynamics on single trials or for estimating the value of small-variance neural signals embedded in the neural population response.

The high spatial resolution offered a number of advantages over sparser sampling, including high-quality single-unit isolation, ability to perform automated drift correction^22,49^ (Extended Data Figs. 4 and 5) and localization of the position and depth of the recording sites within a brain structure (for example, inferring probe depth with respect to cortical lamina), using current source density or other features of the recording. The high density also offered additional potential advances such as identifying putative neuron subclasses using extracellular waveforms^5,10,50^.

Scaling up recordings using the Neuropixels 1.0 NHP probe was straightforward by recording from multiple probes simultaneously. In the present study, we demonstrated recording from up to seven probes and scaling this approach further is realistic. In practice, if many probes were used within a single brain area, the recording yield could be lower than a simple linear scaling, because it can be challenging to optimize the probe placement and insertion geometry for each probe independently. In addition, it was comparatively more complex to prevent any single probe from encountering resistance when transiting the dura. Care should be taken when inserting many probes within a small region, particularly in superficial structures, and the subsequent recordings could exhibit lower neuron yield than anticipated, based on estimates from single-probe recording sessions. Even given this potential for reduced yield, this is still an excellent approach for recording very large populations of neurons.

Deep brain regions have historically received less attention as a result of the relative challenge of recording large numbers of neurons in these areas. The ability to conveniently record from many neurons is transformative, by enabling experiments that would be triaged otherwise, such as high-risk, high-reward projects, or simply additional control experiments. In addition to convenience, however, simultaneous large-scale population recordings from multiple structures make it possible to address questions that would not be possible otherwise, such as identifying communication between areas, for example, via communication subspaces^51,52^, or accurately estimating neural state on single trials^53^.

The recordings presented in this report have exhibited lower probe drift (often <10 µm) than recent Neuropixels recordings in humans, which can exhibit hundreds of micrometers of drift^10,11^. These demonstrations were performed in an operating room during neurosurgeries, which involved creating large craniotomies (sometimes ~30–50 mm in diameter) and reflecting the dura to expose the brain. Without the dura or the skull to mechanically stabilize the brain, changes in pressure from cardiac and pulmonary rhythms cause the brain to pulse, often by hundreds of micrometers. This rapid cyclical drift introduces a challenge for drift correction and spike-sorting algorithms, often reducing the neuron yield relative to a stable preparation.

This challenge is largely absent in many rhesus macaque recordings, which are most often performed with the dura intact, and often using a smaller craniotomy (often as small as 3.5 mm, although this size is not essential for enabling stable recordings). For superficial recordings, such as those in the motor cortex, we placed a blunt guide tube on the surface of the dura with gentle compression (~0.5 mm), which eliminated most residual pulsation. Furthermore, animal experiments have the luxury of additional setup time relative to human recordings in the operating room, which must take place within a 15- to 20-min window. In the present study, we began each experiment by waiting for up to an hour as the probe settled in the brain, which reduced the impact of slow drift from the tissue relaxing back after probe insertion. The commercially released version of Neuropixels 1.0 features two linear columns of recording sites, which is expected to further simplify algorithmic drift correction relative to the probe variant demonstrated here, which featured two zig-zag columns of recording sites.

The Neuropixels 1.0 NHP probe is now commercially available and integrates seamlessly with the existing set of community-supported hardware and software tools for Neuropixels probes. The Neuropixels recording system is straightforward to set up and integrate with other experimental hardware, such as behavior or stimulus control computers. Combining the low total system cost (roughly US$7,000–15,000) and large-scale recordings enables a dramatic reduction in the recording cost per neuron acquired relative to existing technologies.

Although highly capable, these probes are limited in several ways. First, this technology has not been optimized for simultaneous, dense sampling across a wide swath of cortex. For applications requiring horizontal sampling, planar recording technologies like the Utah arrays or 2P imaging may be more appropriate. Second, in contrast with many passive electrodes, it is not currently possible to use the Neuropixels probe to deliver intracortical microstimulation (ICMS), although future versions of the probe may add this functionality. The Neuropixels technology is, however, capable of recording while stimulating through external electrodes, as recently demonstrated by O’Shea et al.^15^. Third, the Neuropixels 1.0 NHP design is not explicitly optimized for chronic implantation. Although it is probably possible to leave the probe in place over multiple days or sessions, this possible capability remains untested and requires new implant designs. The probe base contains active electronics and is not designed for implantation under dura. As such, a chronic implant design may require mounting the probe in a manner that allows it to mechanically ‘float’ with the brain, to prevent relative motion between the probe and the tissue as the brain moves. As such, this probe is most appropriate for acute recordings, although it could conceivably be implanted for subchronic (multiple-week) recordings with appropriate insertion methods and hardware. Last, although it is theoretically possible to insert the entire 45-mm-long shank into the brain, inserting a probe this deep introduces additional practical challenges to overcome—primarily a requirement for precise alignment of the probe’s insertion axis with the insertion location.

Taken together, these methodological advances enable new classes of neuroscientific experiments in large animal models and provide a viable scaling path toward recording throughout the whole brain.

## Methods

All surgical and animal care procedures were performed in accordance with National Institutes of Health (NIH) guidelines and approved by the institutional animal care and use committees of each institution involved in the study, including Stanford University, University of California, Berkeley and Columbia University.

### Probe design and recording system

The Neuropixels 1.0 NHP probe consists of an integrated base and ‘shank’ fabricated as a monolithic piece of silicon using a 130-nm CMOS lithography process. The 6 mm × 9-mm base is mounted to a 7.2 × 23-mm^2^ printed circuit board (PCB), which is attached to a 7.2 × 40-mm^2^-long flexible PCB. This flexible PCB plugs into a ZIF connector on a headstage (15 × 16 mm^2^, 900 mg), which is connected to a PXIe controller mounted in an PXIe chassis using a 5-m twisted-wire cable. The base electronics, headstage, cable, PXIe system and software are identical to the Neuropixels 1.0 probe. Data collection was performed using SpikeGLX software (https://billkarsh.github.io/SpikeGLX/) and the system is fully compatible with OpenEphys software.

The commercial release of these probes features recording sites distributed in two aligned vertical columns, in contrast to the ‘zig-zag’ columns described in the present report, to optimize data collection for automated drift correction and enhance automated spike sorting. A third variant, identical to Neuropixels 1.0 in length, but with a thicker shank (122 µm versus 25 µm), is also now commercially available on special request.

Recording sites are 12 µm × 12 µm, made of titanium nitride and have an impedance of ±150 kΩ at 1 kHz. The tips of the probes were mechanically beveled to a 25° angle using the Narishige EG-402 micropipette beveler. During recordings, electrical measurements were referenced to: (1) the large reference electrode on the tip of the probe; (2) an external electrical reference wire placed within the recording chamber; or (3) a stainless steel guide-tube cannula. Electrical signals are digitized and recorded separately for the action potential band (10 bits, 30 kHz, 5.7 µV mean input-referred noise) and local field potential (LFP) band (10 bits, 2.5 kHz).

Recording sites are programmatically selectable with some constraints on site selection (see Extended Data Fig. 1 for a description of site-selection rules and common configurations). Spike sorting was performed using Kilosort 2.5 and Kilosort 3.0, and results were curated using Phy. Analysis was performed using customized scripts written in Matlab and Python, leveraging the open-source software package neuropixels-utils (https://github.com/djoshea/neuropixel-utils).

### Probe insertion

Several distinct methods were used to mount and insert probes, guided by the unique constraints of inserting probes to different depths and depending on the recording chambers and mechanical access available for different primates used in these studies, as well as the existing hardware used by each of four distinct research groups. For single-probe insertions, probes were mounted using customized adapters to a commercially available probe drive (for example, Narishige, Corp.) and inserted through a blunt guide tube for superficial recordings and a sharp penetrating guide tube for deeper recordings. When using a nonpenetrating guide tube, the dura was typically penetrated with a tungsten electrode before using a Neuropixels probe to create a small perforation in the dura to ease insertion. When inserting probes to deep targets (>20 mm), the alignment between the drive axis and the probe shank is essential for enabling safe insertion, because misalignment can cause the probe to break. For this application, we developed several approaches to maintain precise alignment of the probe and drive axis. First, we employed a linear rail bearing (IKO International) and customized three-dimensional (3D) printed fixture to maintain precise alignment of the insertion trajectory. This approach is discussed in detail in the accompanying Neuropixels 1.0 NHP wiki (https://github.com/cortex-lab/neuropixels/wiki).

For the experiments shown in Fig. 4, we developed a dovetail rail system that maintains precise alignment between a penetrating guide tube and the Neuropixels probe. The choice of appropriate insertion method depends on the mechanical constraints introduced by the recording chamber design, the depth of recording targets, number of simultaneous probes required and choice of penetrating or nonpenetrating guide tube. The interaction of these constraints and a more thorough discussion of insertion approaches are provided on the Neuropixels users wiki (https://github.com/cortex-lab/neuropixels/wiki). Open-source designs for mechanical mounting components for Neuropixels 1.0 NHP to drives from Narishige, NAN and other systems are available in a public repository: https://github.com/etrautmann/Neuropixels-NHP-hardware.

To minimize the impact of slow tissue drift during recordings, the Neuropixels probes were often inserted 150–300 µm past the desired target depth, then withdrawn by that amount and allowed to ‘settle’ for 30–60 min before beginning an experiment. In addition, for superficial recordings in motor cortex, the probe was inserted through a blunt guide tube that was placed in contact with the dural surface and lowered by a small amount (~500 µm), gently compressing the dura to reduce the tissue motion resulting from pulse and respiratory rhythms.

### In vitro electrical characterization

In vitro noise measurements are performed in the standard, self-referenced configuration as described in the Neuropixels manual, with the reference and ground connected together and to a platinum wire electrode in a saline bath. The noise on each channel is measured by averaging Fourier power spectra from 5× 3-s-long sections of data and estimating the root mean square (r.m.s.) from the integral over 300–1,0000 Hz:

$${{\mathrm{r.m.s.}}}=\sqrt{P\times {{\mathrm{Binwidth}}}}=\sqrt{P\times \frac{{f_{\mathrm{s}}}}{{N}_{{{\mathrm{FFT}}}}}}$$

where *P* is the sum over the frequency range in the power spectrum, *f*_s_ the sampling frequency and *N*_FFT_ the number of points in the fast Fourier transform (FFT) calculation. Gain measurements were performed by connecting the probe ground and reference to the Faraday cage ground and a 1-mV, 3-kHz sine wave was applied to the saline bath through a Pt wire electrode. The amplitude of the sine wave on each channel was measured by averaging over a 15-Hz window about the 3-kHz peak in the Fourier power spectrum. Extended Data Fig. 2d shows the mean amplitude over channels versus bank; error bars = 1 s.d.

### Visual cortex recordings and analysis

Two male adult rhesus monkeys (*Macaca mulatta*, 11 and 16 kg), monkey T and monkey H, served as experimental subjects. Each animal was surgically implanted with a titanium head post and a cylindrical titanium recording chamber (30-mm diameter). In each animal, the placement of the recording chamber was centered at ~17 mm from the midline and ~7 mm behind ear-bar-zero, and a craniotomy was performed, allowing access to multiple visual areas in the superior temporal sulcus (STS). All surgeries were conducted using aseptic techniques under general anesthesia and analgesics were provided during post-surgical recovery.

We measured visual RFs by randomly presenting a single-probe stimulus out of either a 7 (H) × 11 (V) stimulus grid extending 18 (H) × 30 (V) degrees of visual angles (d.v.a.) (monkey T) or a 14 (H) × 17 (V) stimulus grid extending 26 (H) × 32 (V) d.v.a. (monkey H). The stimulus consisted of a drifting Gabor gratings (2° in diameter, 0.5 cycle per ° in spatial frequency, 4 ° s^−1^ in speed, 100% Michelson contrast) and was presented for a duration of 0.1 s. Monkeys were rewarded with a drop of juice if they maintained fixating at the fixation spot throughout the trial. Neuropixels data were collected using SpikeGLX. Spike sorting was performed using either Kilosort 2.0 (single-bank recording, monkey T) or Kilosort 3.0 (multi-bank recording, monkey H) and manually curated with Phy. For a given stimulus location, we obtained the neuronal activity by counting all the spikes during the stimulus presentation period, accounted for by a time delay of 50 ms. Neuronal RFs were defined as the stimulus locations that elicited >90% of the peak visual responses.

### Motor cortex recordings and analysis

Details of the pacman behavioral task and experimental hardware presented in Fig. 3 are described in ref. ^33^. Three monkeys (*M. mulatta)* served as experimental subjects. In each, a head post and recording chamber were implanted over premotor and primary motor cortex using aseptic surgical procedures and general anesthesia. Placement of the chambers was guided using structural magnetic resonance imaging (MRI). The recordings in monkey C were performed using a standard 19-mm plastic recording chamber (Christ, Inc.), whereas monkeys I and J were implanted with customized, low-profile, footed titanium chambers (Rogue Research).

We conducted 13 sessions in monkey C and 10 sessions in monkey I, targeting sulcal and gyral M1 and PMd. On a subset of 15 sessions in monkey C, we also targeted GPi in the basal ganglia. In monkey J, we reported data from one session while simultaneously recording in GPi, SMA and M1.

For monkey C, the Neuropixels 1.0 NHP probe was held using a standard 0.25-inch dovetail mount rod with a customized adapter to mount it to a hydraulic drive (Narishige, Inc.). A 21G blunt guide tube, 25 mm in length, was held using a customized fixture and placed over the desired recording location. The dura was then penetrated with a tungsten electrode (FHC, size E), which was bent at 27 mm to prevent the tip from inserting further than 2 mm past the end of the guide tube. This electrode was inserted manually via forceps, once or several times, as necessary, which also provided feedback on the depth and difficulty in penetrating the dura. The Neuropixels 1.0 NHP probe was then aligned using the Narishige tower XY stage, lowered into the guide tube and carefully monitored to ensure that the tip of the probe was aligned with the dural penetration. This procedure sometimes took several attempts to find the correct insertion point, but was generally successful in less than a few minutes.

For monkeys I and J, the Neuropixels 1.0 NHP probe was held using a customized fixture mounted to a linear rail bearing (IKO, Inc.). This apparatus was designed to enable close packing of many probes and to solve the challenge of precisely targeting structures deep in the brain without trial and error. The linear rail is mounted in a customized, 3D printed base, which mounts directly to the recording chamber. The geometry of the 3D printed base component determines the insertion trajectories and prevents mechanical interference between the probes and the chamber. This base also provides support for either sharp or blunt guide tubes, as required. In general, blunt guide tubes were preferred, but if necessary sharp guide tubes were sometimes used when the dura had become thicker and difficult to penetrate. The linear bearing was connected to a commercial drive system (NAN, Inc.) via a ~50-mm-long, 508-mm stainless steel wire, which provided rigid connection between the Nan drive probe mount and the Neuropixels probe mounted on the rail bearing, while allowing a small amount of misalignment between the drive axis and the insertion axis. This apparatus greatly simplifies the procedure of using many probes in a small space, while not relying on commercial drives to provide the mechanical rigidity required to safely insert a delicate probe. Additional details on the customized hardware are provided in the Neuropixels 1.0 NHP user wiki: https://github.com/etrautmann/Neuropixels-NHP-hardware.

Spike sorting was performed using Kilosort 2.5 and manually curated using Phy. Principal component analysis trajectories were calculated after smoothing spikes with a 25-ms Gaussian kernel and averaging across successful trials. Rastermap was run with default parameters after normalizing trial-averaged neural activity. An offline force model prediction performance was computed using a 50-ms time lag between arm force and neural activity. Neurons were randomly subselected and 80% of trials from six target conditions were used to train a linear regression model in Python, using scikit-learn, whereas the remaining 20% of trials were used to calculate model performance. Ten iterations were performed for each level of neurons retained. Probe drift calculations were performed using KS 2.5.

### LIP recordings and analysis

Details of the collection and analysis of the data presented in Fig. 7 are described in ref. ^44^. Two monkeys (*M. mulatta*, 8–11 kg) served as experimental subjects. In each, a head post and two recording chambers were implanted using aseptic surgical procedures and general anesthesia. Placement of the LIP chamber was guided by structural MRI. The SC chamber was placed on the midline and angled back 38° from vertical in the anterior–posterior axis. The decision task is a dynamic, random dot motion (RDM) discrimination task. A schematic of the task is displayed in Fig. 7a and its description can be found in the corresponding figure legend. A second (control) task—an oculomotor delayed response task—was used to measure the response fields of neurons in LIP and SC, described in the legend for Fig. 7c.

We conducted eight recording sessions in which activity in the LIP and SC was recorded simultaneously. In the LIP we used a single Neuropixels 1.0 NHP probe, yielding 54–203 single units per session. In the SC, we used 16-, 24- and 32-channel V-probes (Plexon) with 50- to 100-µm electrode spacing, yielding 13–36 single units per session. In each session, we first lowered the SC probe and approximated the RFs of SC neurons using a few dozen trials of a delayed saccade task. As our penetrations were approximately normal to the retinotopic map in the SC, the RFs of the SC neurons were highly similar within a session. We proceeded only if the center of the RFs was at least 7° eccentric to ensure minimal overlap with the motion stimulus.

If the RF locations in the SC were suitable, we then lowered the Neuropixels probe into the LIP through a dura-penetrating, stainless steel guide tube (23G) at 5 µm s^−1^ using a MEM microdrive (Thomas Recording) that was attached to a chamber-mounted, three-axis micromanipulator. Custom-designed adapters were used for mounting the Neuropixels probe on to the drive (wiki: https://github.com/etrautmann/Neuropixels-NHP-hardware). Once the target depth was reached (~10 mm below the dura), we allowed 15–30 min of settling time to facilitate recording stability. To precisely measure RF locations in both areas, the monkeys performed 100–500 trials of the delayed saccade task and LIP neurons with RFs that overlapped those of the SC neurons were identified post-hoc. Finally, the monkeys performed a reaction-time RDM discrimination task until satiated (typically 1,500–3,000 trials).

Neurons in both areas were sorted using Kilosort 2.0 and manually curated in Phy. We restricted our analysis of the LIP data to neurons with RFs that overlapped those of the simultaneously recorded SC neurons (164 of 1,084 total LIP neurons). Spike trains were discretized into 1-ms bins and convolved with a Gaussian kernel (*σ* = 25 ms) to produce the single-trial activity traces depicted in Fig. 7d,e.

### Face-patch recordings and analysis

Two monkeys (*M. mulatta*) served as experimental subjects. Each animal was surgically implanted with an MRI-compatible Ultem head post and a large rectangular recording chamber (61 at 46-mm diameter, 65 at 50-mm diameter and 58 at 61-mm diameter, respectively), covering most of the animal’s acrylic implant. Monkeys were trained to passively fixate on a spot for juice reward while visual stimuli of 5° size, such as images of faces or objects, were presented on a liquid crystal display (LCD) screen (Acer). We targeted face patches ML and AF (monkey 1) and face-patch AM (monkey 2) in the IT cortex for electrophysiological recordings. Face patches were identified using functional (f)MRI. Monkeys were scanned in a 3T scanner (Siemens), as described previously^56^. MION contrast agent was injected to increase the signal:noise ratio. During fMRI, monkeys passively viewed blocks of faces and blocks of other objects to identify face-selective patches in the brain. During electrophysiology, monkeys viewed two stimulus sets: one consisting of the same stimuli shown during fMRI, consisting of faces and nonface objects, with 150 ms of ON time and 150 ms of OFF time (Fig. 4c,e), and a stimulus set consisting of a monkey face alternating with a lemon every second, without any blank period (Fig. 4b).

Before targeting fMRI-identified face patches with Neuropixels probes, we performed scout recordings with tungsten electrodes with 1-MΩ impedance (FHC) using grids designed with the software Planner^57^. While inserting tungsten electrodes, we performed structural MRI to confirm correct targeting (Fig. 4a). Subsequently, we performed a total of 72 Neuropixels insertions. To perform very deep recordings (for example, 42 mm from the craniotomy; Fig. 4d), we lowered a cannula holder to touch or gently push the dura. The cannula holder contained a short cannula to penetrate the dura. A probe holder, which held the probe, was slid through the cannula holder via matching dovetails. This dovetail mechanism was designed to ensure that the direction of probe movement matched the direction of the cannula, because even small differences in angles would risk breakage of the probe when inserted deeply into the cannula. The probe holder was advanced using an oil hydraulic micromanipulator (Narishige), but, importantly, the precise direction of probe movement was constrained by the dovetail between the probe holder and cannula holder rather than the micromanipulator.

Neuropixels data were recorded using SpikeGLX and OpenEphys and spikes were sorted using Kilosort 3.0. To compute responses for Fig. 4c,e, average spike rates from 50 ms to 250 ms after trial onset were computed and baselines, averaged from 0 ms to 50 ms after trial onset, were subtracted.

### Spike–spike correlation analysis

Functional interactions between pairs of neurons were measured with an established crosscorrelation method. CCGs were calculated using spike trains from pairs of simultaneously recorded neurons, during either the whole stimulus presentation period or the intertrial intervals. The CCG is defined as:

$${{\mathrm{CCG}}}(\tau )=\frac{\frac{1}{M}{\sum }_{i=1}^{M}{\sum }_{t=1}^{N}{x}_{1}^{i}(t){x}_{2}^{i}(t+\tau )}{\theta (\tau )\sqrt{{\lambda }_{1}{\lambda }_{2}}}$$

where *M* is the number of trials, *N* the number of time bins within a trial, ${x}_{1}^{i}$ and ${x}_{2}^{i}$ the spike trains of neurons 1 and 2 from trial *i*, *t* is time, *τ* the time lag relative to the reference spikes and ${\lambda }_{1}$ and ${\lambda }_{2}$ the mean firing rate of the two neurons, respectively. $\theta (\tau )$ is a triangular function calculated as $\theta (\tau )=N-\left|\tau \right|$ which corrects for the overlapping time bins at different time lags. A jitter-corrected method was used to remove correlations caused by stimulus locking or slow fluctuations:

$${{{\mathrm{CCG}}}}_{{{\mathrm{jitter}}\; {\mathrm{corrected}}}}={{{\mathrm{CCG}}}}_{{{\mathrm{original}}}}-{{{\mathrm{CCG}}}}_{{{\mathrm{jittered}}}}$$

where CCG_original_ and CCG_jittered_ are CCGs calculated using the above equation, with the original dataset and the dataset with spike timing randomly perturbed (jittered) within the jitter window, respectively. The correction term (CCG_jittered_) captured slow correlation longer than the jitter window (caused by common stimulation or slow fluctuation in the population response), so, once it’s subtracted, only the fine temporal correlation is preserved. A 25-ms jitter window was chosen based on previous studies^6^. Only well-isolated single units with a firing rate of at least 1 Hz were included for CCG.

As with previous studies^6,13^, in the present study a CCG is classified as significant if the peak of jitter-corrected CCG occurred within 10 ms of zero time lag and if this peak is >7 s.d. values above the mean of the noise distribution (CCG flank).

Neuron centroid positions were estimated by computing the 2-d average of the channel locations of channels on which the spike appears (defined by the Kilosort spatial template for that unit), weighted by the amplitude of the spike on each channel. This calculation is performed using the neuropixel-utils library available online (https://github.com/djoshea/neuropixel-utils).

### Statistics and reproducibility

The main purpose of the present report is to demonstrate the technical viability of a new recording technology. For many of the analyses presented here, standard statistical tests were used. The spike–timing correlation analyses used standard approaches that were used in refs. ^6,13^. For all experiments, animals were trained on the task before any recording. Eight adult male *M. mulatta* animals were used in the results presented here, with data presented for two animals for each of the four example use cases (multiple visual areas, M1, LIP and IT cortex). No statistical method was used to predetermine sample size for each experiment.

The Investigators were not blinded to allocation during experiments and outcome assessment, but the analysis was largely automated, reducing the influence of subjective judgments. In most cases, blinding was not relevant to the technical proofs of concept that we present in this report. Spike sorting was conducted primarily using automated spike-sorting algorithms (Kilosort 2.5 and 3.0), followed by some minimal manual curation and inspection of cluster isolation quality. This manual step was performed by different individuals in different labs, but none of the results presented here are highly sensitive to the individual choices made during spike sorting.

For most experiments, datasets from specific sessions were excluded from further analysis if large-scale probe movement precluded automated spike sorting with Kilosort, but this happened only occasionally and was not a frequent occurrence. The stability recording results presented in Figs. 3–5 are intentionally not statistical arguments, because the stability of any given recording is much more highly determined by the details of the experimental prep and the condition of the implant and tissue. The fraction of datasets with poor recording quality or excessive drift is probably not generalizable to other preparations. Instead, our purpose was to illustrate that highly stable recordings are achievable in multiple brain areas with relative ease. Similarly, we make no statistical claims about the expected yield of neurons recorded on a probe in a given session; instead we present several observed values from these datasets. We feel that this is important because accurate neuron counts remain subjective if manual curation is performed.

### Reporting summary

Further information on research design is available in the Nature Portfolio Reporting Summary linked to this article.

## Online content

Any methods, additional references, Nature Portfolio reporting summaries, source data, extended data, supplementary information, acknowledgements, peer review information; details of author contributions and competing interests; and statements of data and code availability are available at 10.1038/s41593-025-01976-5.

## Supplementary information

Reporting Summary

## Data Availability

Data to replicate key analyses shown in Figs. 1–6 and 8 are available via Zenodo at 10.5281/zenodo.14744139 (ref. ^58^). Data to replicate the analyses of LIP data shown in Fig. 7 are available via Zenodo at https://zenodo.org/records/7946011 (ref. ^59^).

## Code availability

Code to replicate the analysis shown in Fig. 7 is available via Zenodo at https://zenodo.org/records/7946011 (ref. ^59^).
